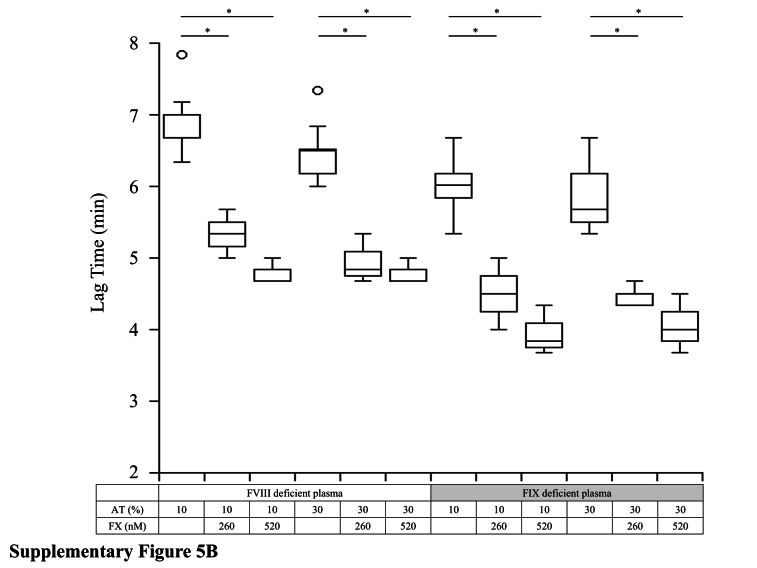# Factor X and combined factor VIIa/factor X augment coagulation potential in a plasma model of antithrombin-reduced hemophilia

**DOI:** 10.1016/j.rpth.2025.103172

**Published:** 2025-09-03

**Authors:** Shigeharu Oh, Yuto Nakajima, Eisuke Takami, Hirotoshi Nakano, Keiji Nogami

**Affiliations:** 1 Department of Pediatrics, Nara Medical University, Kashihara, Nara, Japan; 2 Medical Science of Thrombosis and Bleeding Disorder, Nara Medical University, Kashihara, Nara, Japan; 3 Medical Affairs Section, KM Biologics Co, Ltd, Kumamoto, Japan

**Keywords:** antithrombin, factor VIIa, factor Ⅹa, hemophilia, thrombin

## Abstract

**Background:**

Plasma-derived (pd) factor (F)VIIa/FX products are available for the hemostatic management of people with hemophilia with inhibitors in Japan. We have previously reported that FX alone augments emicizumab-driven hemostasis. Fitusiran is an investigational small interfering RNA that reduces antithrombin (AT) to rebalance hemostasis in people with hemophilia. The effects of supplementation with pd-FVIIa/FX or FX alone on coagulation potential in AT-reduced hemophilia remain to be clarified.

**Objectives:**

To assess the coagulation potential of pd-FVIIa/FX or FX using an *in vitro* model of AT-reduced (fitusiran-treated) people with hemophilia.

**Methods:**

Pd-FVIIa/FX (0.75 and 1.5 μg/mL as FVIIa), recombinant FVIIa (1.1 and 2.2 μg/mL), activated prothrombin complex concentrate (0.65 and 1.3 IU/mL), or FX (260 and 520 nM) were added to FVIII- or FIX-depleted AT-deficient plasmas at AT levels of 10% and 30% (AT-reduced model). Additionally, FX (0-1040 nM) was incubated with FVIII- or FIX-depleted FX-deficient plasmas to assess FVIIa/tissue factor (TF)-mediated FX activation. Coagulation potential was assessed by measuring thrombin generation (TG) and FXa.

**Results:**

The addition of pd-FVIIa/FX, activated prothrombin complex concentrate, or FX to the AT-reduced plasma model of people with hemophilia improved TG potential within the normal range. The addition of recombinant FVIIa, however, had little effect on TG in this model. The addition of FX to FVIII- or FIX-depleted FX-deficient plasma increased TG potential and FVIIa/TF-triggered FXa generation dose-dependently.

**Conclusion:**

Supplementation with FX or pd-FVIIa/FX enhanced FVIIa/TF-induced activation of FX and increased coagulation potential in the AT-reduced plasma model of people with hemophilia.

## Essentials

• Reduction of AT improves the impaired coagulation potential in people with hemophilia.
• The effect of the addition of FVIIa/FX or FX in an AT-reduced hemophilia state is unclear.
• The addition of FX, as well as FVIIa/FX, augmented TG under this condition.
• FX supplementation may be helpful for hemostatic treatment in an AT-reduced hemophilia state.

## Graphical abstract

**Figure:**
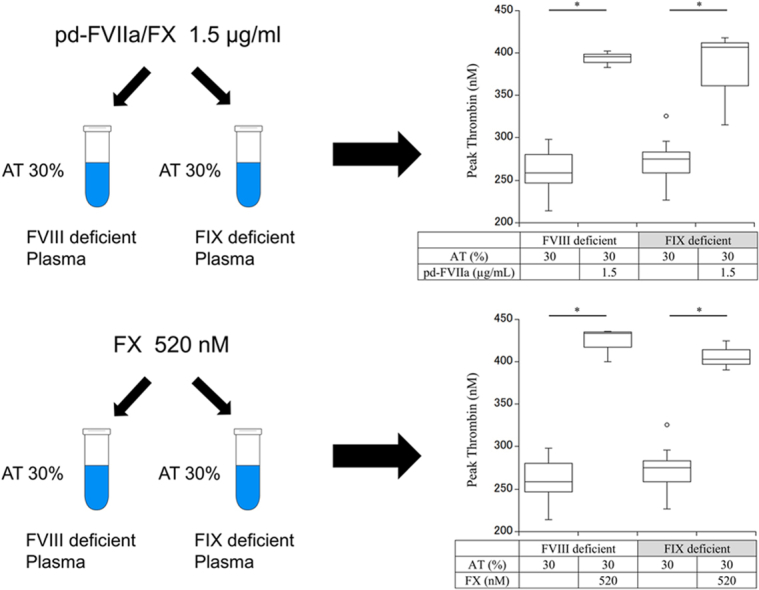

## 1 Introduction

Hemophilia A (HA) and hemophilia B are caused by defects in factor (F)VIII and FIX, respectively, that can lead to uncontrollable bleeding. Anti-FVIII or anti-FIX neutralizing antibodies (inhibitors) develop in 20% to 30% of people with severe HA and in 10% of individuals with severe hemophilia B after repeated infusions of clotting factor concentrates [1,2] and can seriously compromise clinical management [3]. Bypassing agents (BPAs), including recombinant activated FVII (rFVIIa) and activated prothrombin complex concentrate (aPCC), are used for hemostatic therapy in patients with inhibitors [4]. The BPAs are effective but appear to be clinically inconsistent in individual patients, with many factors contributing to the variable responses [5,6].

Consequently, alternative nonfactor product therapies, which rebalance the coagulation status by manipulation of procoagulant or anticoagulant proteins, are being developed. In this context, fitusiran is an investigational small interfering RNA that reduces antithrombin (AT) levels and enhances coagulation potential in people with hemophilia without and with inhibitors. Clinical trials have demonstrated that fitusiran prophylaxis significantly reduces annualized bleeding rates compared with BPAs, although repeated bleeding episodes have been reported in some patients receiving fitusiran [7,8]. Lower doses of concomitant replacement clotting factors or BPAs may be effective in these patients [7]. Thromboembolic episodes have been reported, however, in some people with HA receiving both emicizumab and aPCC [9] and in those receiving fitusiran and rFVIIa [10]. These findings demonstrate that the optimal use of BPAs and nonfactor product therapies, including fitusiran, should be monitored carefully in clinical practice.

A plasma-derived (pd) product consisting of a mixture of FVIIa and FX (pd-FVIIa/FX), at a protein weight ratio of 1:10, was approved for people with congenital hemophilia with inhibitors and acquired hemophilia in Japan in 2014 [11,12], and clinical trials of on-demand and prophylactic pd-FVIIa/FX have been reported [[13], [14], [15]]. In addition, we have shown that supplemental pd-FVIIa/FX could improve coagulation potential in plasmas from people with HA receiving emicizumab prophylaxis [16]. Moreover, our recent study has demonstrated that additional FX alone augments the coagulation potential of emicizumab in *in vitro* experiments using plasma from people with HA and in *in vivo* experiments with HA mice [17]. These data indicate that the use of not only pd-FVIIa/FX but also FX alone might be effective in conjunction with nonfactor products for breakthrough bleeding in people with hemophilia with inhibitors.

Measurements of global coagulation potential, including thrombin generation assays (TGAs), have been developed to assess the clinical use of emicizumab and/or BPAs [18,19]. The present study was therefore designed to investigate hemostasis mechanisms associated with the use of conventional BPAs (rFVIIa and aPCC), pd-FVIIa/FX, or FX in an AT-reduced plasma model (fitusiran-treated) of people with hemophilia.

## 2 Methods

Blood samples from people with HA with inhibitors were collected after informed consent in accordance with the ethical guidelines of Nara Medical University (approval number 2503).

### 2.1 Reagents

rFVIIa (NovoSeven), aPCC (FEIBA), and pd-FVIIa/FX (Byclot) were obtained from Novo Nordisk A/S, Takeda Pharmaceutical Co, Ltd, and KM Biologics Co, Ltd, respectively. Purified pd human FX and anti-FIX polyclonal antibody (polyAb) were obtained from KM Biologics. An anti-FVIII inhibitor polyAb immunoglobulin G was purified from people with HA with inhibitors, as previously described [20]. Recombinant human tissue factor (TF; Innovin), FX-deficient (def) plasma (George King), AT-def plasma (Affinity Biologicals), AT (Prolytix), FX and FXa (Hematologic Technologies), thrombin-specific fluorogenic substrate (Z-Gly-Gly-Arg-AMC, Bachem), thrombin-specific fluorogenic substrate FluCa Kit and thrombin calibrator (Thrombinoscope BV), ellagic acid (Sysmex Corporation), and chromogenic FXa substrate S-2222 (Chromogenix) were purchased from the indicated vendors. PL vesicles containing 10% phosphatidylserine, 60% phosphatidylcholine, and 30% phosphatidylethanolamine were prepared as described previously [21].

### 2.2 Plasma samples

Whole blood samples were collected in plastic tubes containing 3.2% sodium citrate at a ratio of 9:1 (*Fuso Pharmaceutical Industries*). No study subjects had taken any medications that may have influenced platelet or coagulation function 2 weeks prior to blood sampling. Platelet-poor plasma was separated by centrifuging citrated whole blood for 15 minutes at 2000 × *g*. All plasma samples were stored at −80 °C and thawed at 37 °C immediately prior to the assays.

### 2.3 *In vitro* AT-reduced plasma model (fitusiran-treated simulation) of people with hemophilia

The AT-reduced plasma model of people with hemophilia with inhibitors, based on fitusiran treatment, was constituted *in vitro* as follows. AT-def plasma was preincubated with anti-FVIII polyAb or anti-FIX polyAb to completely neutralize FVIII activity (termed “FVIII-depleted”) or FIX activity (termed “FIX-depleted”), as previously described [20]. The BPAs, aPCC (0.65 and 1.3 IU/mL, corresponding to 25 and 50 IU/kg infusion, respectively), and rFVIIa (1.1 and 2.2 μg/mL, corresponding to 45 and 90 μg/kg infusion, respectively) were added to the FVIII-depleted AT-def or FIX-depleted AT-def plasma. Previous studies have indicated that pd-FVIIa/FX infusions at a dose of 60 μg/kg increased FVIIa and FX levels in plasma to 30 to 40 nM and 293 nM, respectively [13,14]. Therefore, in the current experiments, pd-FVIIa/FX at 0.75 and 1.5 μg/mL of FVIIa (corresponding to 30 and 60 μg/kg infusions, respectively) and FX at 260 and 520 nM (corresponding to 2 and 4 IU/mL, respectively) were added to FVIII-depleted AT-def or FIX-depleted AT-def plasmas. Finally, AT (0, 0.24, 0.72, and 2.4 μM corresponding to 0%, 10%, 30%, and 100%, respectively) was added to simulate the AT-reduced plasma (fitusiran-treated) of people with hemophilia.

### 2.4 TGAs

TGA was performed as previously described [19]. Briefly, plasma samples (80 μL) were preincubated for 10 minutes with 20 μL of a trigger reagent containing TF, ellagic acid, and phospholipid vesicles (final concentration 0.5 pM, 0.3 pM, and 4 μM, respectively). After adding 20 μL of a reagent containing CaCl_2_ and fluorogenic substrate (final concentration 16.7 mM and 2.5 mM, respectively), the development of fluorescent signals was monitored using a Fluoroskan Ascent microplate reader (Thermo Fisher Scientific). The thrombin generation curves in all the figures show only the data for the period during which substrate cleavage actively occurred. Data analyses were performed using the manufacturer’s software to derive the standard parameters: peak thrombin (PeakTh) and lag time. The PeakTh and lag time (mean ± SD) obtained from 19 healthy individuals were 501 ± 70 nM and 4.5 ± 0.6 minutes, respectively [16].

### 2.5 FⅩa generation assays

FXa generation assays were performed as described previously [22]. Briefly, FX (0, 32.5, 65, 130, 260, 520, and 1040 nM) in buffer (20 mM HEPES, 0.1 M NaCl, 5 mM CaCl_2_, pH 7.2, 0.01% Tween 20) containing PL vesicles (20 μM) were mixed with rFVIIa/TF (0.1 nM/0.1 nM) and incubated at 37 °C. The reactions were quenched after 5 minutes by adding 50 mM EDTA. Rates of FXa generation were determined after the addition of S-2222 (0.46 mM). The velocity of FXa generation was calculated using a Labsystems Multiskan Multisoft microplate reader (Labsystems).

### 2.6 Statistical analysis

Experiments were performed 3 times, and data were presented as the mean and SD. Data analysis was performed using JMP PRO (JMP Statistical Discovery LLC). Significant differences were determined by the Wilcoxon rank-sum test or Dunnett’s multiple comparison test. *P* values < .05 were considered statistically significant.

## 3 Results

### 3.1 Coagulation potentials of BPAs (rFVIIa, aPCC, or pd-FVIIa/FX) in FVIII- or FIX-depleted AT-reduced plasma

**Supplemental Figure 1:**
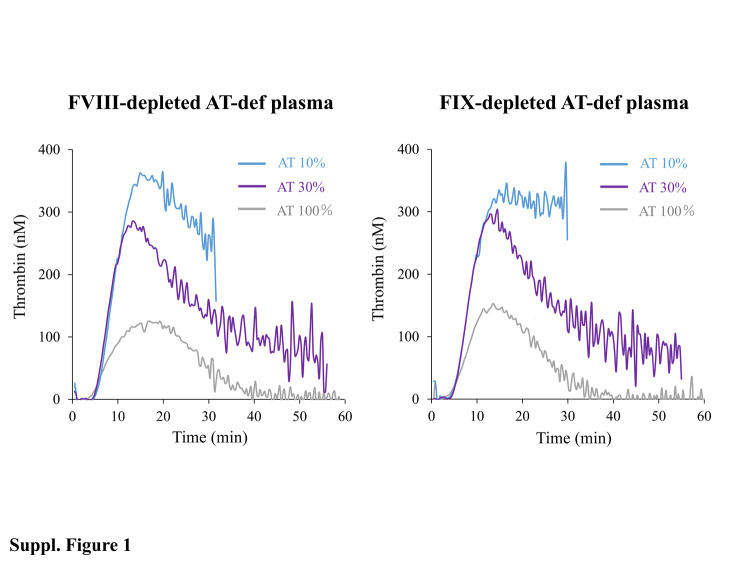
**Thrombin generation in FVIII-depleted or FIX-depleted AT-deficient plasma in the presence of exogenous AT** TF/Elg-triggered thrombin generation was examined in FVIII-depleted or FIX-depleted AT-def plasmas in the presence of various concentrations of AT (10, 30, and 100%): (*blue*, AT 10%; *purple*, AT 30%; *gray*, AT 100%) Abbreviations; AT: antithrombin, AT-def plasma: AT-deficient plasma.

Thrombin generation potential was initially validated in AT-def plasma at different concentrations of AT (0, 0.24, 0.72, and 2,4 μM corresponding to 0%, 10%, 30%, and 100%, respectively). Clinical phase 3 trials of fitusiran treatment indicated that AT levels of 10% and 30% should be considered as the lower and upper targets for fitusiran-induced AT reduction [10], based on the mitigation of thrombotic events. As expected, our results demonstrated that PeakTh was inversely proportional to AT concentrations in AT-def plasma (data not shown). In addition, PeakTh was inversely proportional to the AT concentration in FVIII- or FIX-depleted AT-def plasmas (Supplementary Figure S1). These findings were consistent with an earlier report [23].

**Figure 1:**
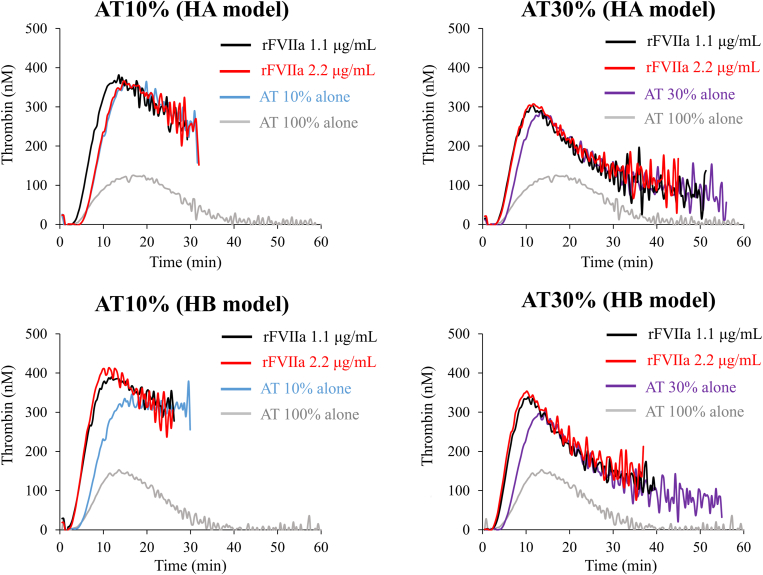
Thrombin generation mediated by recombinant activated factor (F)VII (rFVIIa) in FVIII- or FIX-depleted antithrombin (AT)-deficient plasma in the presence of exogenous AT. FVIII- or FIX-depleted AT-deficient plasma samples were prepared to represent people with hemophilia receiving fitusiran. Tissue factor/ellagic acid-triggered thrombin generation was estimated in the presence of various concentrations of AT (10%, 30%, and 100%). Thrombin generation was monitored after adding exogenous AT together with rFVIIa (1.1 and 2.2 μg/mL). Experiments were performed 3 times, and representative thrombin generation curves are shown (*black*, rFVIIa 1.1 μg/mL; *red*, rFVIIa 2.2 μg/mL; *blue*, AT 10%; *purple*, AT 30%; *gray*, AT 100%). HA, hemophilia A; HB, hemophilia B.

**Figure 2:**
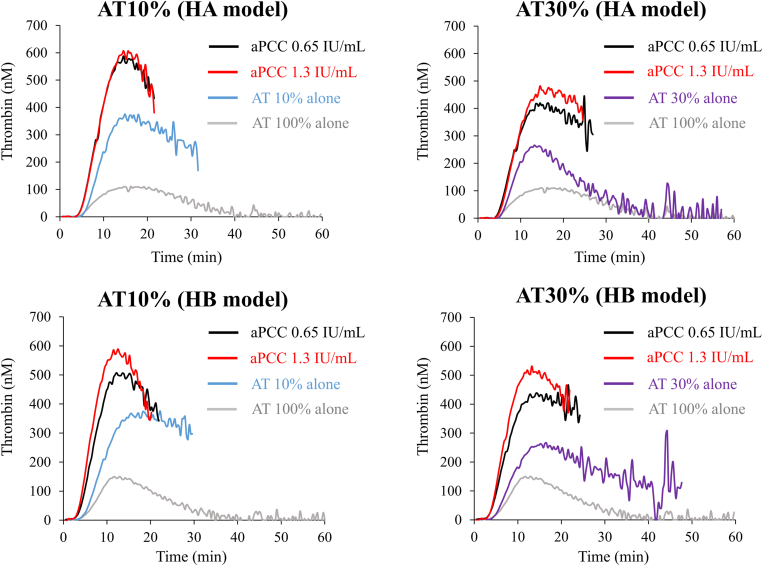
Thrombin generation mediated by activated prothrombin complex concentrate (aPCC) in factor (F)VIII- or FIX-depleted antithrombin (AT)-deficient plasma in the presence of exogenous AT. FVIII- or FIX-depleted AT-deficient plasma was prepared and monitored for tissue factor/ellagic acid-triggered thrombin generation as above. Thrombin generation was estimated in the presence of exogenous AT (10% and 30 %) together with aPCC (0.65 and 1.3 IU/mL). Experiments were performed 3 times, and representative thrombin generation curves are shown (*black*, aPCC 0.65 IU/mL; *red*, aPCC 1.3 IU/mL; *blue*, AT 10%; *purple*, AT 30%; *gray*, AT 100%). HA, hemophilia A; HB, hemophilia B.

**Figure 3:**
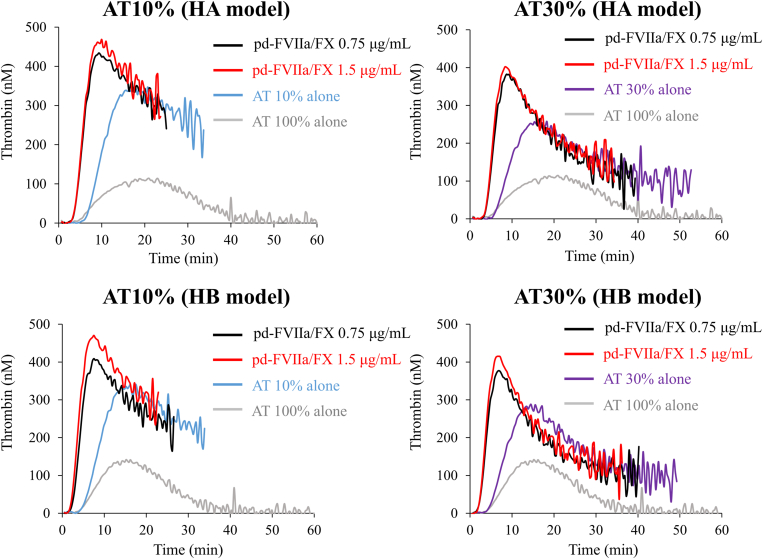
Thrombin generation mediated by plasma-derived activated factor (F)VII (pd-FVIIa)/FX in FVIII- or FIX-depleted antithrombin (AT)-deficient plasma in the presence of exogenous AT. FVIII- or FIX-depleted AT-deficient plasma was prepared and monitored for tissue factor/ellagic acid-triggered thrombin generation as above. Thrombin generation was estimated in the presence of exogenous AT (10% and 30%) together with pd-FVIIa/FX (0.75 and 1.5 μg/mL). Experiments were performed 3 times, and representative thrombin generation curves are shown (*black*, pd-FVIIa/FX 0.75 μg/mL; *red*, pd-FVIIa/FX 1.5 μg/mL; *blue*, AT 10%; *purple*, AT 30%; *gray*, AT 100%). HA, hemophilia A; HB, hemophilia B.

Coagulation potential was further assessed in the presence of BPAs in FVIII- or FIX-depleted AT-reduced plasma. Representative thrombin generation curves are shown in Figure 1, Figure 2, Figure 3. The lag time was shortened in model samples of FVIII- or FIX-depleted AT-reduced plasmas with AT levels of 10% and 30% after the addition of rFVIIa compared with those without rFVIIa. PeakTh levels, however, appeared comparable with those without rFVIIa (Figure 1, Supplementary Figure S2A, B, Supplementary Table S1). The addition of aPCC to these FVIII- or FIX-depleted AT-reduced plasmas also shortened the lag time, but in contrast, PeakTh was significantly increased relative to that in the absence of aPCC and was comparable with the normal level (Figure 2, Supplementary Figure S3A, B, Supplementary Table S1). Similarly, the addition of pd-FVIIa/FX to these model plasma samples significantly shortened the lag time of thrombin generation compared with its absence. The PeakTh was also significantly enhanced in the presence of pd-FVIIa/FX and was comparable or lower than the normal range (Figure 3, Supplementary Figure S4A, B, Supplementary Table S2). These results suggest that these BPAs could enhance coagulation potential in FVIII- or FIX-depleted AT-reduced plasma, and appear unlikely to mediate responses that exceed the normal range.

### 3.2 Coagulation potential of supplementary FX in FVIII- or FIX-depleted AT-reduced plasma

**Figure 4:**
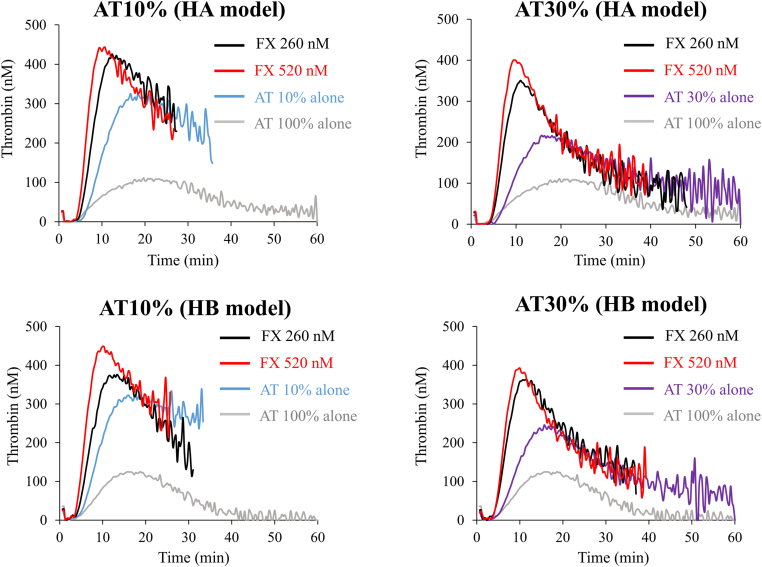
Thrombin generation mediated by factor (F)X in FVIII- or FIX-depleted antithrombin (AT)-deficient plasma in the presence of exogenous AT. FVIII- or FIX-depleted AT-deficient plasma was prepared and monitored for tissue factor/ellagic acid-triggered thrombin generation as above. Thrombin generation was estimated in the presence of exogenous AT (10% and 30%) together with FX (260 and 520 nM). Experiments were performed 3 times, and representative thrombin generation curves are shown (*black*, FX 260 nM; *red*, FX 520 nM; *blue*, AT 10%; *purple*, AT 30%; *gray*, AT 100%). HA, hemophilia A; HB, hemophilia B.

**Supplemental Figure 5:**
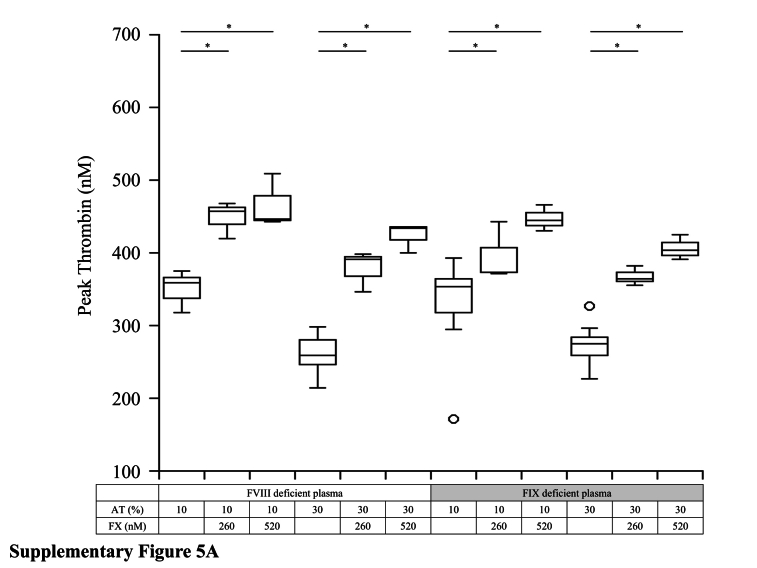
**Thrombin generation in FVIII-depleted or FIX-depleted AT-def plasmas supplemented with AT in the co-presence of FX preparation** TF/Elg-triggered thrombin generation assays (TGA) were performed as described in Methods. The peak thrombin (**A**) and lag time (**B**) obtained from FVIII-depleted AT-def plasmas (HA model) or FIX-depleted AT-def plasmas (HB model) spiked with AT (10% and 30%) and FX preparation (260 or 520 nM) are shown. TG parameters were compared in the absence of BPAs with those in the presence of BPAs. Significant differences were considered as p<0.05 (Wilcoxon rank sum test). Experiments were performed 3 times, and the average values and standard deviation are shown. White circle indicates outlier. The peak thrombin and lag time obtained from 19 healthy individuals were 501±70 nM and 4.5±0.6 min, respectively, as described in Methods. The figures in parenthesis (10), (30) indicate; AT 10%, AT 30%, respectively. Abbreviations; AT: antithrombin, AT-def plasma: AT-deficient plasma, BPA: bypassing agent, HA: hemophilia A, HB: hemophilia B.

The results of our experiments suggested that PeakTh in FVIII- or FIX-depleted AT-reduced plasma in the presence of rFVIIa was similar to that without rFVIIa, but was significantly greater in the presence of aPCC or pd-FVIIa/FX than without these additives (Supplementary Tables S1 and S2). We speculated that FX interactions might especially augment coagulation potential in FVIII- or FIX-depleted AT-reduced plasma. Further experiments were devised to examine the specific effects of FX on coagulation potential in these circumstances. Representative thrombin generation curves are shown in Figure 4. The lag time of FVIII- or FIX-depleted AT-reduced plasma at AT levels of 10% and 30% in the presence of FX was significantly shortened compared with that in its absence. The PeakTh was also significantly increased and was comparable with or lower than normal (Figure 4, Supplementary Figure S5A, B, Supplementary Table S2). The results indicate that supplementary FX could augment coagulation potential within the normal range of FVIII- or FIX-depleted AT-reduced plasma.

### 3.3 Variable coagulation responses in the presence of different BPAs in AT-reduced plasma of people with hemophilia

**Figure 5:**
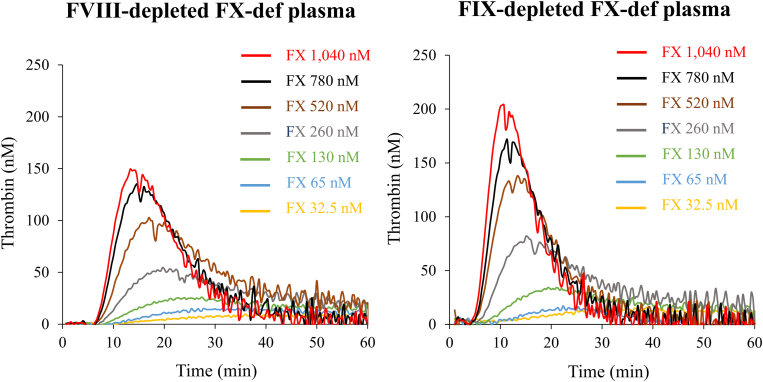
Thrombin generation in factor (F)VIII- or FIX-depleted FX-deficient (def) plasma in the presence of exogenous FX. Tissue factor/ellagic acid-triggered thrombin generation was estimated in FVIII- or FIX-depleted FX-def plasmas in the presence of various concentrations of FX (up to 1040 nM). *(yellow*, FX 32.5 nM; *blue*, FX 65 nM; *green*, FX 130 nM; *gray*, FX 260 nM; *brown*, FX 520 nM; *black*, FX 780 nM; *red*, FX 1040 nM).

Our findings indicated that PeakTh in FVIII- or FIX-depleted AT-reduced plasmas in the presence of rFVIIa was similar to that in its absence, but was significantly increased in the presence of pd-FVIIa/FX or FX (Supplementary Tables S1 and S2). The data suggested that the limited response mediated by rFVIIa might be associated with reduced FX levels in the plasma model of people with HA and acquired HA, as previously reported [24]. In contrast, coagulation potential could be augmented by the enhanced generation of FXa mediated by FVIIa/TF in the presence of pd-FVIIa/FX or FX alone. Further experiments were therefore performed to examine TGA in FVIII- or FIX-depleted FX-def plasma supplemented with various concentrations of FX (up to 1040 nM; Figure 5, Supplementary Table S3). PeakTh levels were increased in a FX dose-dependent manner. The PeakTh of FVIII- or FIX-depleted FX-def plasma was significantly higher with FX at more than 780 and 520 nM, respectively, than with FX at 130 nM (physiological concentration).

### 3.4 Impact of additional FX on FVIIa/TF-induced FXa generation

Our data also demonstrated that thrombin generation in FVIII- or FIX-depleted AT-def plasma was enhanced by FX in FVIIa/TF-induced interactions. This mechanism was further examined using assays of FXa generation mediated by FX in the presence of physiological concentrations of FVIIa/TF. FXa generation was proportionately enhanced by increasing concentration of FX and was significantly greater in the presence of 1040 nM FX than at physiological concentration (130 nM; Table). These results provided additional evidence that supplementary FX augments coagulation potential in AT-reduced plasma of people with hemophilia by mediating FVIIa/TF-induced FXa generation.

**Table:** Factor VIIa/tissue factor-induced factor Xa generation and the effect of various concentrations of factor X.

FⅩ (nM)	32.5	65	130	260	520	1040
Generated FXa (nM)	5.3 ± 0.6	7.9 ± 1.1	11.2 ± 2.4	12.7 ± 1.3	13.7 ± 0.8	15.6 ± 3.0^a^

An FXa generation assay was performed as described in the Methods. Various concentrations of FX (up to 1040 nM), recombinant activated factor VII (0.1 nM), tissue factor (0.1 nM), and phospholipid vesicles (20 μM) were incubated for 5 minutes at 37°C and were added to EDTA-containing tubes to quench the reactions. Rates of FXa generation were determined by the addition of S-2222 (0.46 mM). Generated FX after the addition of various FX amounts, and those after the addition of FX 130 nM (physiological concentration), were compared. Significant differences were considered as *P* < .05 (Dunnett’s test). Experiments were performed 3 times, and the average values and SD are shown.

EDTA, ethylene-diamine-tetraacetic acid; FX, factor X; FXa, activated factor X.

a *P* < .05 vs FX 130 nM.

Overall, our findings were in keeping with the hypothesis that reduced levels of AT impair FXa generation and that therapeutic FX or pd-FVIIa/FX could enhance FVIIa/TF-induced activation of FX and increase coagulation potential in the AT-reduced plasma of people with hemophilia.

## 4 Discussion

A recent report suggested that thrombotic events in people with hemophilia receiving fitusiran prophylaxis were associated with AT levels [10]. In detail, the incident rate of thrombosis per 100 patient-years at AT levels of <10%, 10% to 20%, and >20% was 5.91, 1.49, and 0, respectively. Target AT levels of 15% to 35% were recommended for mitigating thrombotic risk [10]. In this context, the present study was designed to set the AT level at 10% (lower level) and 30% (higher level). Furthermore, a later phase 3 open-label study of fitusiran prophylaxis demonstrated that the average AT level was 9% to 13% [7]; our minimum AT level could provide pertinent information in these circumstances. Our *in vitro* findings suggested that pd-FVIIa/FX products or FX alone, as well as conventional BPAs, could increase coagulation function in FVIII- or FIX-depleted AT-reduced plasma at AT levels of 10% and 30%. These data provide evidence that pd-FVIIa/FX and FX preparations, which are clinically available or under development in Japan (jRCT203124028), could be effective in treating bleeding events in people with hemophilia who are receiving fitusiran.

The current study showed that rFVIIa added to an AT-reduced plasma model of people with hemophilia did not enhance coagulation potential, consistent with a previous report [23]. However, caution is warranted regarding our results because the *in vitro* model of thrombin generation in plasma does not accurately reflect the clinical observations. In the ATLAS-OLE study [25], very small doses of either aPCC or rFVIIa were successfully used to treat bleeds in patients receiving fitusiran. It is likely that the *in vitro* model may be biased toward the other agents because high phospholipids are used as the procoagulant surface. Studies have shown that rFVIIa acts on platelet surfaces [26], which are absent in our study. Therefore, it is likely that different results would have been observed if the study had used whole blood for thrombin generation rather than plasma.

In contrast, the addition of a BPA (aPCC or pd-FVIIa/FX) and FX augmented coagulation function in our AT-reduced plasma model of people with hemophilia. Under these circumstances, the supplementary FX appeared to likely have increased FXa production (see Figure 5, Table). The aPCC products contain clotting factors, such as FIX/FIXa, FX/FXa, and FII/thrombin, which enhance coagulation potential, and our data showed that measurements of thrombin generation were within the normal range in AT-reduced plasma of people with hemophilia in the presence of 25 to 50 IU/kg aPCC. The recommended dose of aPCC is 30 IU/kg in people with hemophilia receiving fitusiran [10], and our results demonstrated that this dose appears to be appropriate. Our experiments further indicated that increasing concentrations of FX could enhance FVIIa/TF-induced coagulation reactions in the AT-reduced plasma model of people with hemophilia (see Figure 5, Table).

Thrombotic events have been identified in people with hemophilia receiving fitusiran prophylaxis concomitantly with rFVIIa [10]. The precise mechanism(s) remain unclear; however, a fatal thrombotic event with rFVIIa occurred when a previous study [10] was targeting lower AT levels, and the patient was given a large amount of rFVIIa to treat the suspected bleed. Therefore, the use of rFVIIa in patients receiving fitusiran, when administered according to clinical guidelines, is not associated with a significant risk of thrombosis.

The affinity of FVIIa for FX has been estimated at a *K*_m_ (Michaelis-Menten constant) of 250 nM in the presence of PL and 205 nM in the presence of TF with PL [27]. The physiological concentration of FX is approximately 130 nM. In the present context, increased FX concentrations possibly promote FVIIa/TF-induced FX activation to FXa, and consequently, the additive or synergistic effect on FVIIa/TF-induced FX activation may contribute to augment hemostatic potential in fitusiran-treated people with hemophilia. We previously demonstrated that additional FX also enhances coagulation potential in the presence of emicizumab [17], indicating that FX, in combination with various nonfactor therapies, would contribute to effective management of people with hemophilia in difficult clinical circumstances. Further studies are required, however.

There are some limitations of the present study. Clinical trials are ongoing, and confirmatory *ex vivo* experiments using plasma from people with hemophilia receiving fitusiran prophylaxis have not been performed. Nevertheless, the addition of exogenous AT to FVIII- or FIX-depleted AT-def plasmas appeared to provide a suitable fitusiran plasma model, and our TGA results appeared to be similar to findings of other fitusiran clinical studies [23]. In addition, the *in vitro* model of thrombin generation in plasma does not always align with clinical observations because rFVIIa is effective in treating breakthrough bleeding in people with hemophilia who receive fitusiran in clinical situations [25,28].

In conclusion, FX and pd-FVIIa/FX augment thrombin generation in FVIII- or FIX-depleted AT-def plasmas in the presence of AT by promoting FVIIa/TF-induced FX activation. Hence, not only the pd-FVIIa/FX product but also FX preparations may be useful for breakthrough bleeding in people with hemophilia receiving fitusiran prophylaxis. Careful monitoring of coagulation potential would be required for the use of BPAs for breakthrough bleeding in people with hemophilia receiving fitusiran prophylaxis.

## Figures and Tables

**Supplemental Figure 2:**
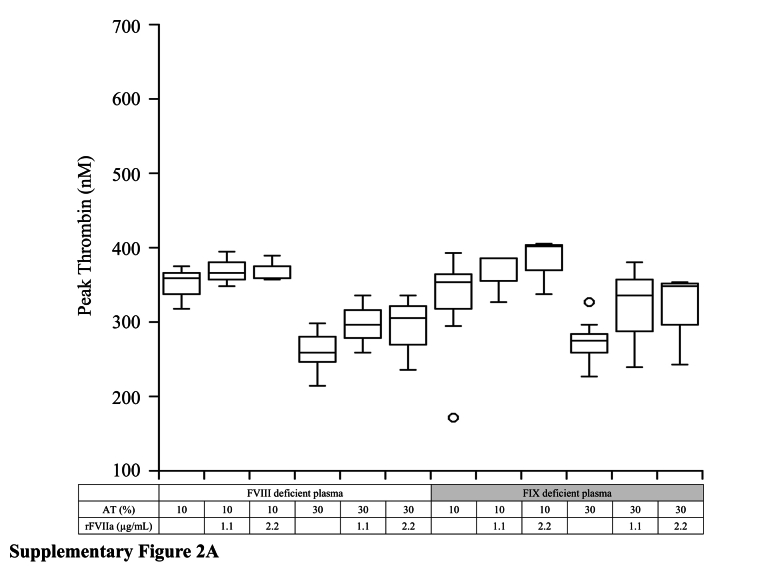
**Thrombin generation in FVIII-depleted or FIX-depleted AT-def plasmas supplemented with AT in the co-presence of rFVIIa** TF/Elg-triggered thrombin generation assays (TGA) were performed as described in Methods. The peak thrombin (**A**) and lag time (**B**) obtained from FVIII-depleted AT-def plasmas (HA model) or FIX-depleted AT-def plasmas (HB model) spiked with AT (10% and 30%) and rFVIIa (1.1 and 2.2 μg/mL) are shown. TG parameters were compared in the absence of BPAs with those in the presence of BPAs. Significant differences were considered as p<0.05 (Wilcoxon rank sum test). Experiments were performed 3 times, and the average values and standard deviation are shown. White circle indicates outlier. The peak thrombin and lag time obtained from 19 healthy individuals were 501±70 nM and 4.5±0.6 min, respectively. The figures in parenthesis (10), (30) indicate; AT 10%, AT 30%, respectively. Abbreviations; AT: antithrombin, AT-def plasma: AT-deficient plasma, BPA: bypassing agent, HA: hemophilia A, HB: hemophilia B.

**Supplemental Figure 2B:**
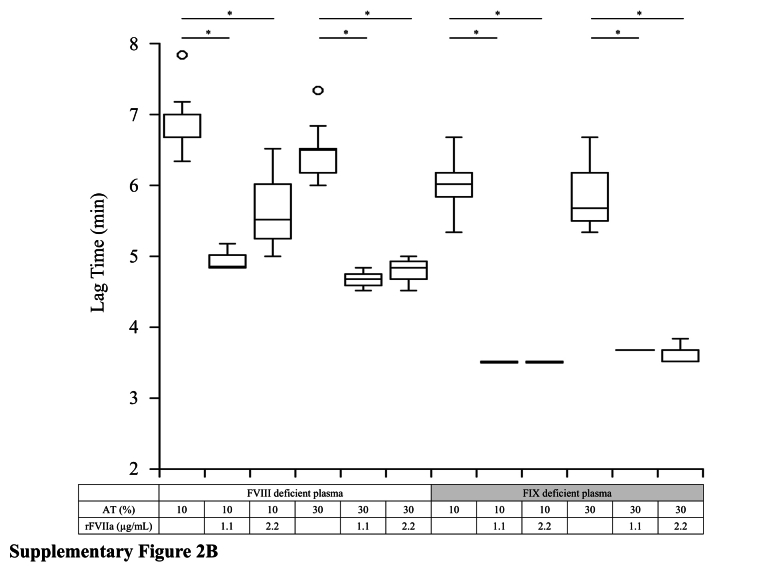


**Supplemental Figure 3:**
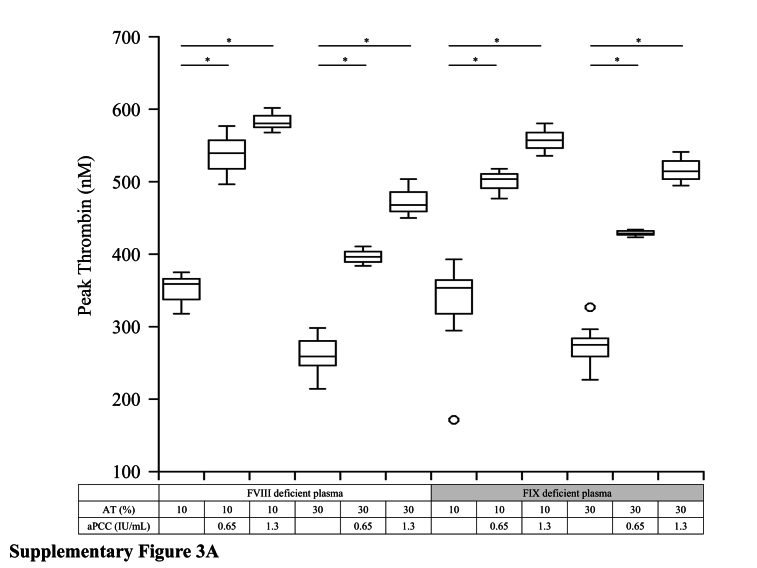
**Thrombin generation in FVIII-depleted or FIX-depleted AT-def plasmas supplemented with AT in the co-presence of aPCC** TF/Elg-triggered thrombin generation assays (TGA) were performed as described in Methods. The peak thrombin (**A**) and lag time (**B**) obtained from FVIII-depleted AT-def plasmas (HA model) or FIX-depleted AT-def plasmas (HB model) spiked with AT (10% and 30%) and aPCC (0.65 and 1.3 IU/mL) are shown. TG parameters were compared in the absence of BPAs with those in the presence of BPAs. Significant differences were considered as p<0.05 (Wilcoxon rank sum test). Experiments were performed 3 times, and the average values and standard deviation are shown. White circle indicates outlier. The peak thrombin and lag time obtained from 19 healthy individuals were 501±70 nM and 4.5±0.6 min, respectively. The figures in parenthesis (10), (30) indicate; AT 10%, AT 30%, respectively. Abbreviations; AT: antithrombin, AT-def plasma: AT-deficient plasma, BPA: bypassing agent, HA: hemophilia A, HB: hemophilia B.

**Supplemental Figure 3B:**
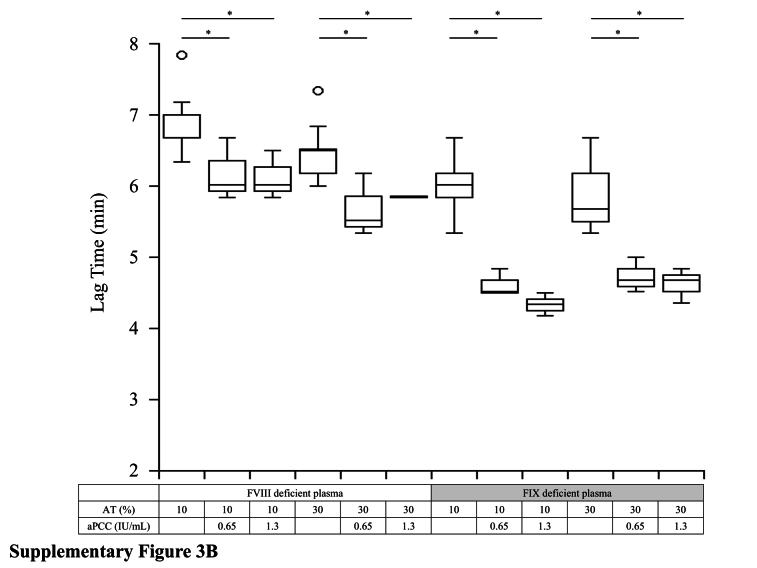


**Supplemental Figure 4:**
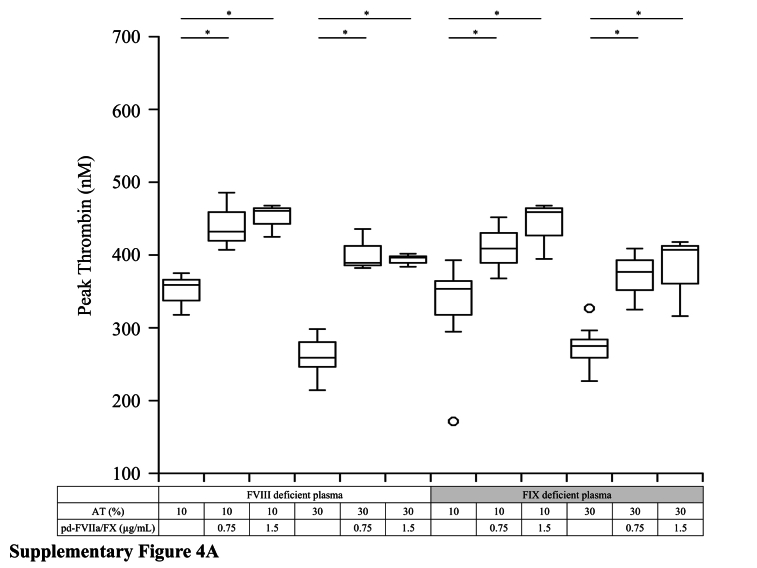
**Thrombin generation in FVIII-depleted or FIX-depleted AT-def plasmas supplemented with AT in the co-presence of pd-FVIIa/FX** TF/Elg-triggered thrombin generation assays (TGA) were performed as described in Methods. The peak thrombin (**A**) and lag time (**B**) obtained from FVIII-depleted AT-def plasmas (HA model) or FIX-depleted AT-def plasmas (HB model) spiked with AT (10% and 30%) and pd-FVIIa/FX (0.75 or 1.5 μg/mL) are shown. TG parameters were compared in the absence of BPAs with those in the presence of BPAs. Significant differences were considered as p<0.05 (Wilcoxon rank sum test). Experiments were performed 3 times, and the average values and standard deviation are shown. White circle indicates outlier. The peak thrombin and lag time obtained from 19 healthy individuals were 501±70 nM and 4.5±0.6 min, respectively. The figures in parenthesis (10), (30) indicate; AT 10%, AT 30%, respectively. Abbreviations; AT: antithrombin, AT-def plasma: AT-deficient plasma, BPA: bypassing agent, HA: hemophilia A, HB: hemophilia B.

**Supplemental Figure 4B:**
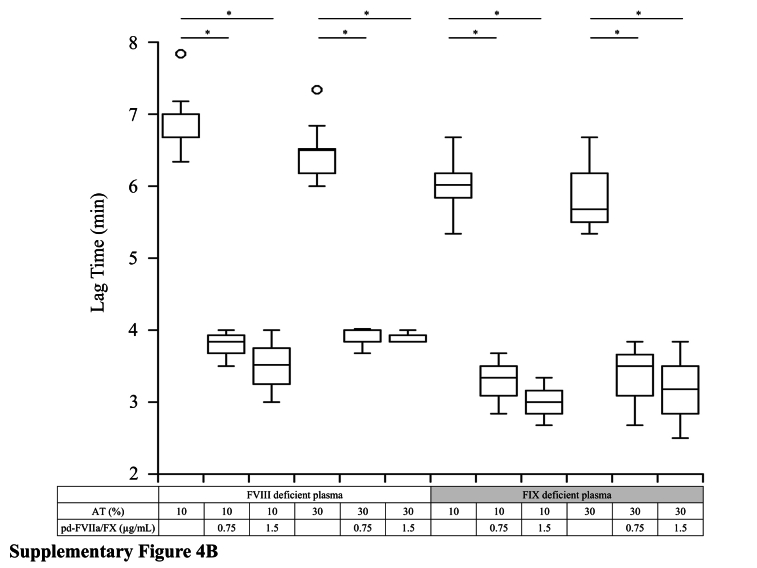


**Supplemental Figure 5B:**