# Supplementary material for: Factor X and combined factor VIIa/factor X augment coagulation potential in a plasma model of antithrombin-reduced hemophilia
Source: Res Pract Thromb Haemost. 2025 Sep 3;9(6):103172. doi: 10.1016/j.rpth.2025.103172 (PMC12495158; doi:10.1016/j.rpth.2025.103172)
Supplement: Supplementary material [file mmc1.docx]

**Supplemental Table 1. Parameters in FVIII-depleted or FIX-depleted AT-def plasmas supplemented with AT in the co-presence of rFVIIa or aPCC**

TF/Elg-triggered thrombin generation assays (TGA) were performed as described in Methods. The parameters obtained from FVIII-depleted AT-def plasmas (HA model) or FIX-depleted AT-def plasmas (HB model) spiked with AT (10% and 30%) and rFVIIa (1.1 and 2.2 µg/mL) or aPCC (0.65 and 1.3 IU/mL) are shown. TG parameters were compared in the absence of BPAs with those in the presence of BPAs. Significant differences were considered as p <0.05 (Wilcoxon rank sum test). Experiments were performed 3 times, and the average values and standard deviation (SD) are shown. The peak thrombin (PeakTh) and lag time obtained from 19 healthy individuals were 501±70 nM and 4.5±0.6 min, respectively as described in Methods. The figures in parenthesis (10), (30) indicate; AT 10%, AT 30%, respectively. Abbreviations; AT: antithrombin, AT-def plasma: AT-deficient plasma, BPA: Bypassing agent, HA: Hemophilia A, HB: Hemophilia B, PeakTh: peak thrombin.

| FVIII-depleted AT-def plasma  (HA model) | PeakTh | Lag time |  | FIX-depleted AT-def plasma  (HB model) | PeakTh | Lag time |
| --- | --- | --- | --- | --- | --- | --- |
|  | *nM* | *min* |  |  | *nM* | *min* |
| AT (10) alone | 351 ± 18 | 6.8 ± 0.4 |  | AT (10) alone | 333 ± 56 | 6.0 ± 0.4 |
| AT (30) alone | 261 ± 25 | 6.4 ± 0.4 |  | AT (30) alone | 272 ± 25 | 5.8 ± 0.4 |
| AT (10) / rFⅦa 1.1 µg/mL | 370 ± 23 | 5.0 ± 0.2* |  | AT (10) / rFⅦa 1.1 µg/mL | 366 ± 34 | 3.5 ± 0.0* |
| AT (10) / rFⅦa 2.2 µg/mL | 369 ± 18 | 5.7 ± 0.8* |  | AT (10) / rFⅦa 2.2 µg/mL | 382 ± 38 | 3.5 ± 0.0* |
| AT (30) / rFⅦa 1.1 µg/mL | 297 ± 39 | 4.7 ± 0.2* |  | AT (30) / rFⅦa 1.1 µg/mL | 318 ± 72 | 3.7 ± 0.0* |
| AT (30) / rFⅦa 2.2µg/mL | 292 ± 52 | 4.8 ± 0.2* |  | AT (30) / rFⅦa 2.2 µg/mL | 315 ± 62 | 3.7 ± 0.0* |
| AT (10) / aPCC 0.65 IU/mL | 537 ± 40* | 6.2 ± 0.4* |  | AT (10) / aPCC 0.65 IU/mL | 499 ± 20* | 4.6 ± 0.2* |
| AT (10) / aPCC 1.3 IU/mL | 583 ± 17* | 6.1 ± 0.3* |  | AT (10) / aPCC 1.3 IU/mL | 557± 22* | 4.3 ± 0.2* |
| AT (30) / aPCC 0.65 IU/mL | 396 ± 14* | 5.7 ± 0.4* |  | AT (30) / aPCC 0.65 IU/mL | 429 ± 5* | 4.7 ± 0.2* |
| AT (30) / aPCC 1.3 IU/mL | 474 ± 27* | 5.8 ± 0.0* |  | AT (30) / aPCC 1.3 IU/mL | 516 ± 24* | 4.6 ± 0.2* |

* p <0.05 *vs* no BPA

**Supplemental Table 2. Parameters in FVIII-depleted or FIX-depleted AT-def plasmas supplemented with AT in co-presence of pd-FVIIa/FX or FX preparation**

TF/Elg-triggered thrombin generation assays (TGA) were performed as described in Methods. The parameters obtained from FVIII-depleted AT-def plasmas (HA model) or FIX-depleted AT-def plasmas (HB model) spiked with AT (10 and 30 %) and pd-FVIIa/FX (0.75 or 1.5 µg/mL) or FX preparation (260 or 520 nM) are shown. TGA parameters were compared in the absence of pd-FVIIa/FX or FX preparation with those in the presence of pd-FVIIa/FX or FX preparation. Significant differences were considered as p <0.05 (Wilcoxon rank sum test). Experiments were performed 3 times, and the average values and standard deviation (SD) are shown. The peak thrombin and lag time obtained from 19 healthy individuals were 501±70 nM and 4.5±0.6 min, respectively as described in Methods. The figures in parenthesis (10), (30) indicate; AT 10%, AT 30%, respectively. Abbreviations; AT: antithrombin, AT-def plasma: AT-deficient plasma, HA: Hemophilia A, HB: Hemophilia B, PeakTh: peak thrombin.

| FVIII-depleted AT-def plasma  (HA model) | PeakTh | Lag time |  | FIX-depleted AT-def plasma  (HB model) | PeakTh | Lag time |
| --- | --- | --- | --- | --- | --- | --- |
|  | *nM* | *min* |  |  | *nM* | *min* |
| AT (10) alone | 351 ± 18 | 6.8 ± 0.4 |  | AT (10) alone | 333 ± 56 | 6.0 ± 0.4 |
| AT (30) alone | 261 ± 25 | 6.4 ± 0.4 |  | AT (30) alone | 272 ± 25 | 5.8 ± 0.4 |
| AT (10) / pd-FⅦa/FⅩ 0.75 µg/mL | 441 ± 40* | 3.8 ± 0.3* |  | AT (10) / pd-FⅦa/FⅩ 0.75 µg/mL | 410 ± 43* | 3.3 ± 0.4* |
| AT (10) / pd-FⅦa/FⅩ 1.5 µg/mL | 447 ± 20* | 3.6 ± 0.5* |  | AT (10) / pd-FⅦa/FⅩ 1.5 µg/mL | 449 ± 37* | 2.9 ± 0.3* |
| AT (30) / pd-FⅦa/FⅩ 0.75 µg/mL | 402 ± 29* | 3.9 ± 0.2* |  | AT (30) / pd-FⅦa/FⅩ 0.75 µg/mL | 370 ± 42* | 3.3 ± 0.6* |
| AT (30) / pd-FⅦa/FⅩ 1.5 µg/mL | 396 ± 9* | 3.8 ± 0.1* |  | AT (30) / pd-FⅦa/FⅩ 1.5 µg/mL | 388 ± 49* | 3.0 ± 0.6* |
| AT (10) / FⅩ 260 nM | 448 ± 25* | 5.3 ± 0.3* |  | AT (10) / FⅩ 260 nM | 395 ± 40* | 4.5 ± 0.5* |
| AT (10) / FⅩ 520 nM | 466 ± 38* | 4.8 ± 0.2* |  | AT (10) / FⅩ 520 nM | 446 ± 18* | 3.9 ± 0.3* |
| AT (30) / FⅩ 260 nM | 378 ± 28* | 4.9 ± 0.3* |  | AT (30) / FⅩ 260 nM | 367 ± 14* | 4.4 ± 0.2* |
| AT (30) / FⅩ 520 nM | 423 ± 20* | 4.8 ± 0.2* |  | AT (30) / FⅩ 520 nM | 406 ± 17* | 4.1 ± 0.4* |

* p <0.05 *vs* no pd-FVIIa/FX or FX

**Supplemental Table 3. Parameters in FVIII-depleted or FIX-depleted FⅩ-deficient plasmas added various concentrations of FⅩ preparation**

TF/Elg-triggered thrombin generation assays (TGA) were performed as described in Methods. The parameters obtained after the addition of FⅩ (up to 1,040 nM) to FVIII-depleted FⅩ-def plasmas (HA model) or FIX-depleted FⅩ-def plasmas (HB model) are shown. TGA parameters in FVIII-depleted or FIX-depleted FX-def plasma spiked with different FX concentrations and those spiked with FX preparation 130 nM were compared. Significant differences were considered as p <0.05 (Dunnett’s test). Experiments were performed 3 times, and the average values and standard deviation (SD) are shown. Abbreviations; HA: Hemophilia A, HB: Hemophilia B, PeakTh: peak thrombin.

| FVIII-depleted FX-def plasma  (HA model) | PeakTh | Lag time |  | FIX-depleted FX-def plasma  (HB model) | PeakTh | Lag time |
| --- | --- | --- | --- | --- | --- | --- |
|  | *nM* | *min* |  |  | *nM* | *min* |
| FⅩ 32.5 nM | 12 ± 8 | 13.1 ± 2.1 |  | FⅩ 32.5 nM | 48 ± 30 | 10.1 ± 0.9 |
| FⅩ 65 nM | 26 ± 22 | 11.6 ± 1.9 |  | FⅩ 65 nM | 56 ± 36 | 9.3 ± 1.0 |
| FⅩ 130 nM | 40 ± 36 | 9.4 ± 0.9 |  | FⅩ 130 nM | 84 ± 44 | 7.2 ± 0.7 |
| FⅩ 260 nM | 68 ± 34 | 8.9 ± 1.0 |  | FⅩ 260 nM | 126 ± 41 | 6.5 ± 0.5 |
| FⅩ 520 nM | 108 ± 33 | 8.3 ± 0.8 |  | FⅩ 520 nM | 176 ± 38* | 5.9 ± 0.5 |
| FⅩ 780 nM | 139 ± 30* | 8.0 ± 0.5 |  | FⅩ 780 nM | 209 ± 36* | 5.6 ± 0.3 |
| FⅩ 1,040 nM | 155 ± 22* | 8.0 ± 0.3 |  | FⅩ 1,040 nM | 238 ± 43* | 5.5 ± 0.2 |

* p <0.01 *vs* FX 130 nM
